# *Epichloë bromicola* from wild barley improves salt-tolerance of cultivated barley by altering physiological responses to salt stress

**DOI:** 10.3389/fmicb.2022.1044735

**Published:** 2022-11-24

**Authors:** Zhengfeng Wang, Jing Liu, James F. White, Chunjie Li

**Affiliations:** ^1^Economic Crops and Malt Barley Research Institute, Gansu Academy of Agricultural Science, Lanzhou, China; ^2^State Key Laboratory of Herbage Improvement and Grassland Agro-Ecosystems, Key Laboratory of Grassland Livestock Industry Innovation, Ministry of Agriculture and Rural Affairs, Engineering Research Center of Grassland Industry, Ministry of Education, Gansu Tech Innovation Center of Western China Grassland Industry, Centre for Grassland Microbiome, College of Pastoral Agriculture Science and Technology, Lanzhou University, Lanzhou, China; ^3^Department of Plant Biology, Rutgers University, New Brunswick, NJ, United States; ^4^Grassland Research Center of National Forestry and Grassland Administration, Chinese Academy of Forestry Sciences, Beijing, China

**Keywords:** *Epichloë* endophyte, barley, salt tolerance, biomass, metabolomics

## Abstract

**Introduction:**

*Epichloë bromicola* is a cultivable fungal endophyte that lives in symbiosis with wild barley (*Hordeum brevisubulatum*) to which it confers salt tolerance. This study tested the hypothesis that *E. bromicola* derived from wild barley has the potential to increase salt tolerance in cultivated barley under salt stress.

**Methods:**

To test this hypothesis, the growth response, physiological parameters, and metabolic profiles of barley plants inoculated with *E. bromicola* (E+) and those not inoculated with *E. bromicola* (E–) were compared under salt stress.

**Results:**

Compared with E– barley plants, E+ barley plants had significantly increased plant height, shoot biomass, total biomass, chlorophyll content, osmotic synthesis, and accumulation of stress adaptation metabolites. *E. bromicola* increased the salt stress tolerance of cultivated barley, and the positive effects correlated with different salt stress conditions.

**Discussion:**

These results suggest that *E. bromicola* has promising potential for enhancing the salt tolerance of barley. New insights into the mechanisms underlying this barley–fungal endophyte association are provided, and interesting questions regarding the role of *E. bromicola* in fungus-enhanced tolerance to salt stress in this symbiosis are raised.

## Introduction

Salt stress is one of the main environmental factors that limiting plant growth and crop yields worldwide (Munns, 2002; Acuña-Rodríguez et al., 2019). Excess irrigation is a major cause of salt deposits in soils of cropland (Habib et al., 2016). Because of the resulting soil salinity, a large area of arable land is being abandoned every year (Chen H. Y. et al., 2021). Salt stress affects up to 20% of the Earth's total cultivated land (Cheng et al., 2012), and 32 million hectares of dryland agriculture is estimated to be affected by excess salt (FAO, 2015). Soil salinization of cultivated land poses an increasing challenge for crop cultivation. Early studies have demonstrated that abiotic stress alone can reduce global crop yields by more than half that would be achievable under optimal growth conditions (Wang et al., 2022).

While barley (*Hordeum vulgare* L.) is the most important salt-tolerant cereal crop, if it is grown in highly saline soils, yields still decrease. At present, traditional practices to breed barley combined with improved salt tolerance have made certain progress (Kuchel et al., 2006), but conventional breeding approaches do not making use of the complex ecological potential of growth-promoting microorganisms in plants. Endophytes of the genus *Epichloë* are microorganisms that infect plants and live in symbiosis with host plants, where they inhabit the aboveground parts of the plant, usually expressing no apparent symptoms in the host plant (Baltruschat et al., 2008; Lugtenberg et al., 2016). *Epichloë* endophytes promote plant growth (Hall et al., 2014; Li et al., 2021; Liu et al., 2022) and improve plant resistance to drought and salt stress (Song et al., 2015; Chen T. X. et al., 2021), as well as resistance to certain pathogens and insects (Moate et al., 2012; Xia et al., 2018). In return, host plants provide nutrients, a spatial structure suitable for habitation, and transmission to the next generation of the plant (Aly et al., 2010).

Plant–endophyte associations may play important roles in grassland agricultural ecology. *Epichloë* endophytes are transmitted vertically through seeds following colonization of the embryo (Christensen et al., 2008). The fungi can be easily isolated from the host and cultured in the laboratory (Bacon and White, 1994). Using these cultures, selected plant–endophyte associations can be obtained through artificial inoculation, resulting in novel cultivars. In addition, artificial transmission of *Epichloë* endophytes from one plant to another can be performed in the laboratory (Bacon and White, 1994), enabling the creation of desirable grass–endophyte combinations (Johnson et al., 2013).

Many studies have shown the benefits of *Epichloë* endophytes in agricultural systems; however, most studies focused on major pasture grass species (Johnson et al., 2013; Le Cocq et al., 2017; Kenyon et al., 2019). For example, several *Epichloë* endophyte strains (i.e., MaxQ^®^, MaxP^®^, AR1, and AR37) have been used to artificially inoculate tall fescue and perennial ryegrass with similar benefits, which have been successfully commercialized in New Zealand, South America, Australia, and the United States (Johnson et al., 2013; Young et al., 2013; Lugtenberg et al., 2016). Many methods of *Epichloë* endophyte inoculation have been employed, including infection of mature tillers, infection of callus cultures, and infection of wounds (Simpson and Mace, 2012). The “seedling wounding: method for inoculation with selected endophytic fungi forms the basis for exploiting *Epichloë* endophytes commercially (Latch and Christensen, 1985; Johnson et al., 2013). To date, *Epichloë* endophyte inoculation of modern cereal grasses has not been reported (Simpson et al., 2014; Yi et al., 2018). Simpson and Mace (2012) found that inoculation of *Epichloë* species from primary hosts to new hosts is more successful when host plants are phylogenetically close. Based on this finding, new combinations can be made that are fully functional in pastoral agricultural systems (Easton et al., 2001).

Plant metabolites may play an important role in regulating the salt stress response of various plant species. However, the molecular mechanism of metabolic changes induced by associations of *Epichloë bromicola* and barley under salt stress remains poorly understood. Non-targeted metabolomics examines all metabolites, thus serving as a bridge between phenotypic and genotypic groups. Non-targeted metabolomics are also an excellent tool for identifying thousands of metabolites that are potentially linked to the regulation of plant and fungal interactions, and it is especially useful for pathway analyses (Schliemann et al., 2008). Previous studies have investigated plant and microbe interactions in the fungal endophyte *Piriformospora indica* colonizing Chinese cabbage roots (Hua et al., 2017), in arbuscular mycorrhizal fungi colonizing ragwort (Hill et al., 2018), and in mycorrhizal fungi colonizing a species of orchid (Ghirardo et al., 2020). Moreover, metabolomics is a very important approach for studying the salt stress response mechanism of plant–fungus interactions (Ashar et al., 2022). Metabolomics aids the study of metabolic pathways in response to stressors (Luo et al., 2009).

Previous studies have examined the effects of *E. bromicola* on wild barley (*Hordeum brevisubulatum*) under salt stress, and it has been well demonstrated that *E. bromicola* plays a key role in the adaptation of wild barley to saline environments. For example, *E. bromicola* can enhance the growth of wild barley, modify its nutrient stoichiometry, adjust ionic homeostasis, and enhance the conversion of putrescine to spermidine and spermine (Song et al., 2015; Chen T. X. et al., 2021). In addition, Rodriguez et al. (2008) found that endophytes collected from coastal habitats can colonize monocots to which they confer habitat-specific salt tolerance. *E. bromicola* isolated from wild barley may hold the potential to confer salt stress tolerance to cultivated barley. Regarding the use of *E. bromicola*, better knowledge and understanding of how the inoculation of barley plants with *E. bromicola* (E+) increases the tolerance of barley against salt stress is essential. This article addresses the question whether *E. bromicola* derived from wild barley has the potential to increase the salt stress resistance of cultivated barley. Based on prior studies on wild barley, it was predicted that endophyte-infected barley would exhibit greater biomass, higher chlorophyll content, and increased capacity for osmotic synthesis compared with endophyte-free plants. Increases in the relative water content of leaves and decreases in reactive oxygen membrane injury rate under salt stress were also predicted. The compositions of metabolites of E+ and E– (not inoculated with *E. bromicola*) plants under different salt stress conditions were compared by a non-targeted liquid chromatograph mass spectrometry (LC-MS) metabolome analysis.

## Materials and methods

**Plant and endophyte:** Barley is widely cultivated in China as it is the most salt-tolerant cereal crop. Barley seeds were sourced from the Gansu Academy of Agricultural Sciences (Lanzhou, China). The fungal endophyte WBE1 was isolated from wild barley (*H. brevisubulatum*) from Gansu, China, and was identified as *E. bromicola* based on its morphological and phylogenetic characteristics analyzed by (Chen T. X. et al., 2018). WBE1 was selected because it can confer salt stress tolerance (Wang et al., 2019; Chen T. X. et al., 2021) to the wild barley association and produce peramine and chanoclavine I; however, loline, lolitrem B, and complex ergot alkaloids are not produced (Chen et al., 2019b).

**Seedling inoculation and endophyte detection:** Fungal endophyte isolates were obtained from wild barley using potato dextrose agar culture media and grown for 14 days to ensure that mycelia are young and vital. Endophytes were then inoculated into barley seedings following the protocols of Latch and Christensen (1985) and Saikkonen et al. (2010). Barley seeds were surface-sterilized by immersing them for 3 min in 70% ethanol, followed by 5 min in 1% NaClO, and then washing with sterilized water three times. Surface-sterilized seeds were placed on 3% water agar (weight/volume) for germination at 22°C in the dark. At 3–5 days after germination, the seedings were inoculated by making an incision near the meristematic tissue and then inserting mycelia into the area of incision. Control seedlings were inoculated with agar. The inoculated seedlings were maintained in a growth chamber at 24°C in the dark for 7 days, followed by a 12/12-h light/dark regimen for 7 days, following the procedures reported in Oberhofer and Leuchtmann (2014). The seedlings were transplanted into plastic pots containing heat-sterilized vermiculite (150°C for 24 h) and maintained in a growth chamber (12/12-h light/dark regime, 24°C). One leaf sheath per plant was sampled by microscopic examination 30 days after transplantation (Bacon and White, 1994). At seed maturity, three seeds were sampled per plant to test whether previously the inoculated endophytes survived in offspring seeds of every inoculated barley plant (Saha et al., 1988). The endophytes were isolated from the seeds, and their morphological characteristics and DNA sequences were analyzed, as described in Chen et al. (2019b).

**Salt treatment:** Barley seeds with and without endophytes were obtained; three seeds each of E+ and E– were sown in separate plastic pots that contained heat-sterilized vermiculite (150°C for 24 h), and the seedlings were irrigated with water as needed. The presence of endophytes was monitored microscopically, using stain 0.8% aniline blue throughout the experiments. After 60 days, sets of E+ and E– plants were bottom-watered with sterile water containing 0, 100, or 300 mM NaCl in the greenhouse (environmental conditions: 22/15°C light/dark cycle, relative humidity 65%, 800 μmol m^−2^ s^−1^ photon flux density, and a photoperiod of 14 h). After 21 days of NaCl treatment, sampling for analyses was performed. The pot experiment involved 3 salt stress levels (0, 100, or 300 mM NaCl) ×2 endophyte types (with and without endophyte) ×3 replicates (pots) ×5 plants per pot, resulting in a total of 90 plants.

**Plant biomass and physiological measurements:** Plant height was measured in pots using a ruler; then, plants were removed from pots, and roots were separated from shoots and washed in sterile water to remove attached soil. Both shoots and roots were weighed to obtain the fresh weight. The chlorophyll content was estimated according to the procedures described by He et al. (2018). In brief, 0.1 g of fresh leaf samples were extracted in 80% acetone and 95% alcohol in the dark and centrifuged at 9,000 rpm for 10 min at 4°C; then, the absorbance was read at 645 and 663 nm to assess chlorophyll a and chlorophyll b contents, respectively. The relative water content (RWC) of leaves was calculated as RWC = (FW – DW) × (TW – DW) – 1 × 100%, where FW is the fresh weight, DW is the dry weight, and TW is the turgid weight (leaves were placed in the dark for 24 h in vials containing water, permitting complete rehydration), following the method of Gulen and Eris (2003). Relative electrical conductivity (REC) was measured as described by He et al. (2018). REC (%) was calculated by (S1/S2) × 100, where S1 and S2 are the electric conductivity values of fresh leaves and boiled leaves, respectively. The proline content was determined following Tiwari et al.'s (2016) method. The absorbance of the supernatant was measured at 525 nm using a spectrophotometer. Finally, the proline concentration was calculated using a calibration curve and was expressed as μg proline g^−1^ FW. The soluble sugar content was determined using anthrone colorimetry, as described by Leakey et al. (2009). The absorbance of the supernatant was measured at 620 nm using a UV spectrophotometer (SP-723). The soluble protein content from leaves was extracted using the G250-Coomassie brilliant blue method by following the method of Bian et al. (2018).

**Sample preparation for metabolite analysis:** A total of six leaves of the same part from different plants were collected from five individual plants and pooled to create one biological sample. Overall, six biological samples were created, and all samples were flash-frozen in liquid N^2^ and stored at −80°C. The leaves (50 mg) from E+ and E– plants in the salt stress experiment were extracted using 800 μL of precooled extraction reagent [MeOH:H_2_O (70:30, v/v, precooled at −20°C)], containing 20 μL of internal standards (d3-leucine, 13C9-phenylalanine, d5-tryptophan, and 13C3-progesterone). Then, two small steel balls were added to the Eppendorf tube and then disrupted with TissueLyser (50 Hz, 5 min, JXFSTPRP, China). The leaves were sonicated for 30 min at 4°C and incubated at −20°C for 1 h. The leaf homogenate was centrifuged at 14,000 rpm for 15 min at 4°C. The supernatants (600 μL) were filtered through 0.22-μm microfilters and collected in autosampler vials for LC-MS analysis. Before leaf sample analyses, 20 μL of the supernatant was prepared for a quality control (QC) sample from each sample to evaluate the reproducibility and stability of the LC-MS analysis.

**Metabolite analysis by LC-MS:** The samples were analyzed using a Waters ACQUITY UPLC 2D (Waters, USA), coupled to a Q-Exactive mass spectrometer (Thermo Fisher Scientific, USA) with a heated electrospray ionization source. The analysis conditions of chromatography are summarized as follows: Hypersil GOLD aQ column (2.1^*^100 mm, 1.9 μm, Thermo Fisher Scientific, USA) with mobile phase A consisting of 0.1% formic acid in water and mobile phase B consisting of 0.1 formic acid in acetonitrile, temperature 40°C, the gradient c 5% B for 0.0–2.0 min, 5–95% B for 2.0–22.0 min, held constant at 95% B for 22.0–27.0 min, washed with 95% B for 27.1–30 min, the flow rate 0.3 mL/min, and the injection volume 5 μL. The analysis conditions of mass spectrometry are as follows: spray voltage, 3.8–3.2 kV; aux gas flow rate, 10 arbitrary units (arb); aux gas heater temperature, 350°C; sheath gas flow rate, 40 (arb); and capillary temperature, 320°C. The full scan was acquired as 100–1,500 m/z with a resolution of 70,000, and the automatic gain control target for MS acquisitions was set to 1e6 with a maximum ion injection time of 100 ms. The top three precursors were chosen for subsequent MSMS fragmentation with a maximum ion injection time of 50 ms, a resolution of 30,000, and automatic gain control of 2e5. The stepped normalized collision energy was set to 20, 40, and 60 eV.

**MS data and statistical analyses:** Non-targeted metabolomics analysis was performed using LC-MS. Data were acquired in positive-ion mode to improve the metabolite coverage using a *Q* Exactive HF (Thermo Fisher Scientific, USA) high-resolution mass spectrometer. LC-MS data were processed using Compound Discoverer 3.1 (Thermo Fisher Scientific, USA), which mainly included peak extraction, peak alignment, and metabolite identification. Metabolites were annotated using the BGI Library (Wuhan Metware Biotechnology Co., Ltd.; http://www.metware.cn, Wuhan, China) and mzCloud database. Data preprocessing was carried out using metaX (Wen et al., 2017). The quality of data was evaluated by the repeatability of QC samples, including chromatogram overlap, principal component analysis, peak response intensity difference, and peak number (Guida et al., 2016). Multivariate statistical analysis (PLS-DA), univariate analysis fold change (FC), and Student's *t*-test were used to screen for differential metabolites between groups. To judge the quality of the PLS-DA model, 200 response permutation tests were conducted. Univariate analysis was performed using FC analysis (FC ≥ 1.2 or ≤ 0.83) and *t*-test (Student's *t*-test, *p*-value < 0.05). Kyoto Encyclopedia of Genes and Genomes (KEGG) pathway database was used for the metabolite functional annotation of pathways (http://www.kegg.jp/kegg/pathway.html). Metabolic pathway enrichment analysis of differential metabolites was performed on the Metabolite Sets Enrichment Analysis (http://www.msea.ca) web-based server, and metabolic pathways with a *p*-value < 0.05 were considered significantly enriched.

### Statistical analysis

In addition to the metabolomic analysis of software and methods already described, all data were explored for homogeneity and normality of variances. We evaluated a general linear model to analyze the effects of endophyte infection (E+ and E–), with salt treatments (CK and salt treatment) and their interactions, as fixed effects, on plant height, plant biomass, total chlorophyll content, RWC, REC, and the ability of osmotic synthesis (i.e., proline, soluble sugar, and soluble protein content). Statistical analysis was performed using the SPSS 19.0 statistical program (SPSS Inc., Chicago, IL, USA), and the ANOVAs were presented to evaluate the significance of endophyte treatments, salt treatments, and fixed factors. Tukey's test was used to test whether there was a difference between E+ and E– barley plants. Differences were considered significant at a *p*-value < 0.05.

## Results

### *Epichloë bromicola* inoculation enhances plant growth under salt stress

**Figure 1:**
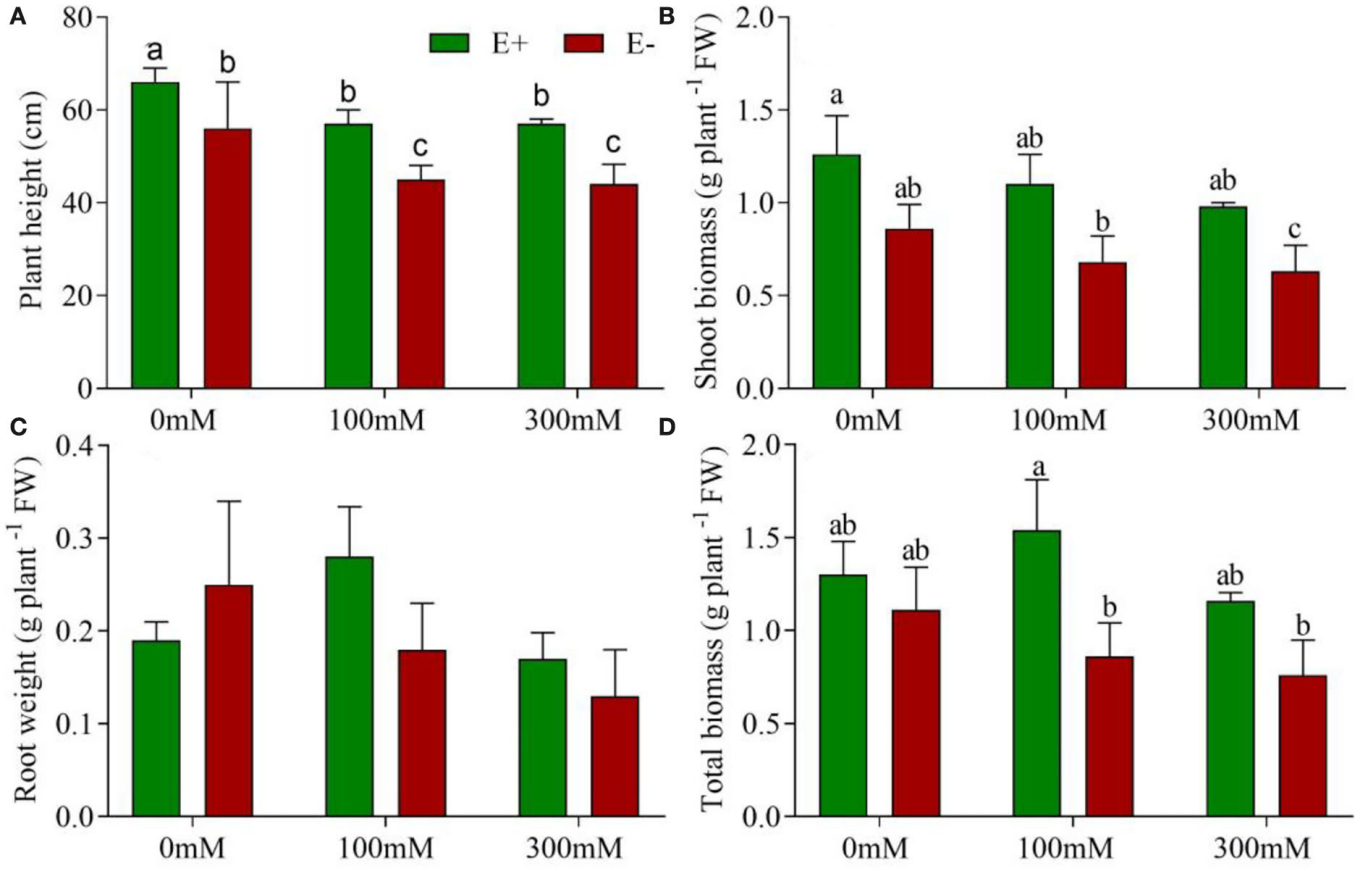
*E. bromicola* enhances barley growth under salt stress conditions. Plant height **(A)**, shoot biomass **(B)**, root biomass **(C)**, and total biomass **(D)** of barley affected by *E. bromicola* inoculation and subjected to 0, 100, and 300 mM NaCl (values are means ± SE). Green bars represent E+ barley, and red bars represent E– barley. Measurements were carried out after 21 days of plant growth under salt stress. Different letters indicate significance at a *p*-value < 0.05 among treatments (Tukey's test). FW = fresh weight.

Endophyte-inoculated barley demonstrated no signs of pathogenesis; rather, endophyte treatment exhibited some positive effects on growth. Plant height was affected by *E. bromicola* and salt (*F* = 59.75, *p* < 0.001, *F* = 20.62, *p* < 0.001, respectively), while it was not significantly affected by *E. bromicola* × salt interaction (*F* = 0.10, *p* = 0.901, Table 1). Importantly, the heights of E– barley plants were, on average, 17.8, 26, and 29% higher than those of E– barley plants at NaCl concentrations of 0, 100, and 300 mM, respectively (Figure 1A). E+ barley plants produced significantly more shoot biomass and total biomass than E– barley plants under several salt treatments (Figures 1B,D). Shoot biomass was significantly affected by *E. bromicola* (*F* = 10.24, *p* = 0.008, Table 1). This was especially apparent at NaCl concentrations of 200 and 300 mM; E+ barley plants had significantly greater shoot biomass (85, 55%) than E– barley (Figure 1B). However, root biomass did not differ between E+ and E– plants (Figure 1C). *E. bromicola*, salt, and *E. bromicola* × salt interaction had no effect on root biomass (*F* = 0.42, *p* = 0.527, *F* = 1.13, *p* = 0.353, *F* = 1.02, *p* = 0.387, respectively). Although no significant effects of *E. bromicola* on root biomass were detected, the root biomass was higher in E+ barley plants than in E-barley plants at all salt levels.

**Table 1:** Analysis of variance for the effects of *E. bromicola* on barley development and physiological response under salt stress conditions.

**Parameter**	**Effect**	***df***	***F***	***P***
Plant height (cm)	E	1,12	59.75	0.000^**^
	S	2,12	20.62	0.000^**^
	E × S	2,12	0.10	0.901^ns^
Shoot biomass (g Plant^−1^ FW)	E	1,12	10.24	0.008^**^
	S	2,12	0.86	0.445^ns^
	E × S	2,12	0.68	0.525^ns^
Root biomass (g Plant^−1^ FW)	E	1,12	0.42	0.527^ns^
	S	2,12	1.13	0.353^ns^
	E × S	2,12	1.02	0.387^ns^
Total biomass (g Plant^−1^ FW)	E	1,12	6.77	0.023^*^
	S	2,12	1.00	0.394^ns^
	E × S	2,12	0.80	0.468^ns^
Root-to-shoot ratio (Plant^−1^ FW)	E	1,12	1.65	0.222^ns^
	S	2,12	0.80	0.469^ns^
	E × S	2,12	0.39	0.684^ns^
RWC (%)	E	1,12	11.85	0.005^**^
	S	2,12	80.65	0.000^**^
	E × S	2,12	0.83	0.457^ns^
REC (%)	E	1,12	27.89	0.000^**^
	S	2,12	144.27	0.000^**^
	E × S	2,12	9.71	0.003^**^
Total chlorophyll (mg g^−1^)	E	1,12	34.80	0.000^**^
	S	2,12	24.44	0.000^**^
	E × S	2,12	1.93	0.187^ns^
Proline (ug g^−1^)	E	1,12	44.55	0.000^**^
	S	2,12	26.81	0.000^**^
	E × S	2,12	93.48	0.000^**^
Soluble sugar (mg g^−1^)	E	1,12	37.46	0.000^**^
	S	2,12	65.93	0.000^**^
	E × S	2,12	7.35	0.008^**^
Soluble protein (mg g^−1^)	E	1,12	147.32	0.000^**^
	S	2,12	69.86	0.000^**^
	E × S	2,12	18.50	0.000^**^

Two-way ANOVA for the endophyte status (E), salt treatment (S), and the interaction E × S for the parameters of plant height, shoot biomass, root biomass, total biomass, root-to-shoot ratio, RWC, REC, total chlorophyll contents, soluble protein contents, protein contents, and soluble sugar contents variables of barley. FW = fresh weight.

** Significant difference p < 0.001,

* significant difference p < 0.05, ns, non–significant.

### *Epichloë bromicola* changes physiological parameters

**Figure 2:**
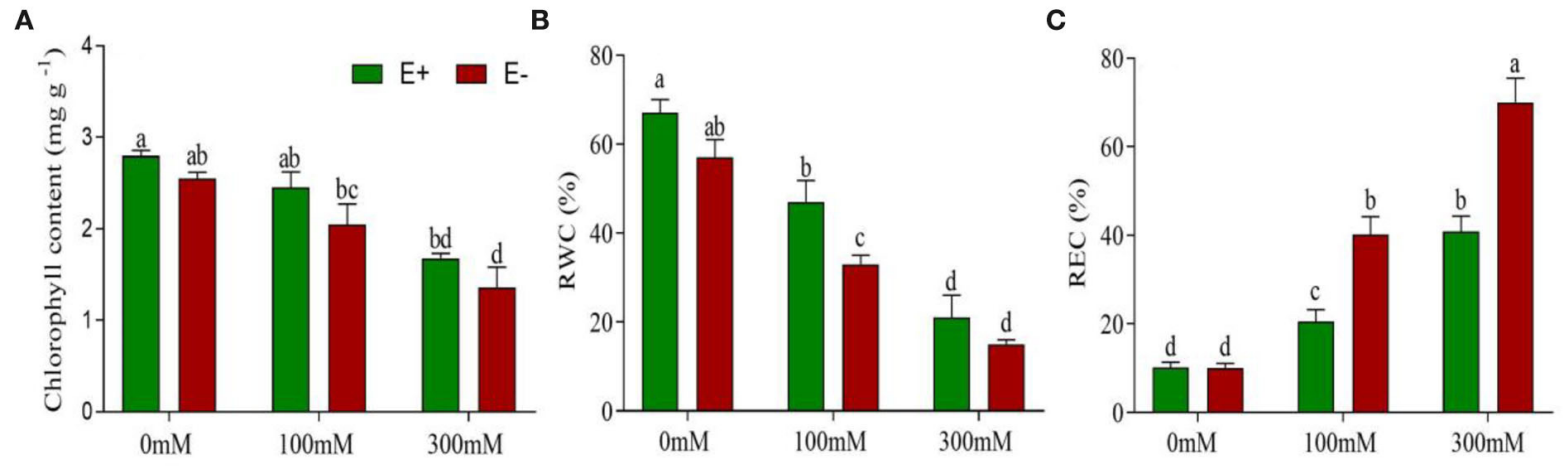
*Epichloë bromicola* increases the leaf chlorophyll content, RWC, and decreases the REC in barley under salt stress conditions. Chlorophyll contents **(A)**, RWC **(B)**, and REC **(C)** of barley inoculated with *E. bromicola* and subjected to NaCl concentrations of 0, 100, and 300 mM (values are means ± SE). Green bars represent E+ barley, and red bars represent E– barley. Measurements were carried out after 21 days of plant growth under salt stress. Different letters indicate significance at a *p*-value < 0.05 among treatments (Tukey's test).

Barley plants treated with NaCl for 21 days suffered from early senescence and exhibited stunted growth. The biomass of young leaves was slightly decreased, while that of older leaves developed yellow necrosis under salt stress. *E. bromicola* inoculation and salt stress had significant effects on the chlorophyll content during the experiment (*F* = 34.80, *p* < 0.001, *F* = 24.44, *p* < 0.001, respectively, Table 1). However, the chlorophyll content did not vary strongly between E+ and E– barley plants under the same salt treatments (Figure 2A).

RWC was significantly affected by *E. bromicola* and salt (*F* = 11.85, *p* = 0.05, *F* = 80.65, *p* < 0.001, respectively, Table 1). E+ barley plants had higher RWC in all treatments. Salt treatment caused a significant decrease in the RWC in both E+ and E– barley plants. However, the effects of *E. bromicola* were more visible at NaCl concentrations of 100 mM between E+ and E– barley plants (Figure 2B).

REC was affected by *E. bromicola* (*F* = 27.89, *p* < 0.001), salt (*F* = 144.27, *p* < 0.001), and their interaction (*F* = 9.71, *p* = 0.003, Table 1). Salt stress led to a significant decrease in REC in both E+ and E– barley. REC variation was evaluated in several different salt treatments, and salt was found to increase plant REC under all conditions. The REC of E+ barley decreased less than that of E– barley at 0 mM NaCl concentrations. This effect was more pronounced for E– barley plants, which produced, on average, 39 and 30% more REC than E+ barley plants at 100 and 300 mM NaCl concentrations, respectively (Figure 2C).

### *Epichloë bromicola* enhances osmotic adjustment capacity under salt stress

**Figure 3:**
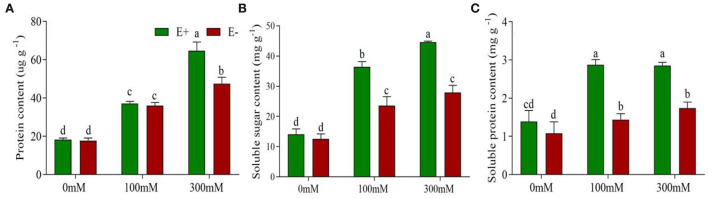
*Epichloë bromicola* enhances barley osmotic adjustment capacity under salt stress conditions. Proline contents **(A)**, soluble sugar contents **(B)**, and soluble protein contents **(C)** of barley inoculated with *E. bromicola* and subjected to NaCl concentrations of 0, 100, and 300 mM (values are means ± SE). Green bars represent E+ barley, and red bars represent E– barley. Measurements were carried out after 21 days of plant growth under salt stress. Different letters indicate significance at a *p*-value < 0.05 among treatments (Tukey's test).

Highly significant effects were found for *E. bromicola* and salt on proline (*F* = 44.45, *p* < 0.001, *F* = 26.81, *p* < 0.001), soluble sugar (*F* = 37.46, *p* < 0.001, *F* = 65.93, *p* < 0.001), and soluble protein contents (*F* = 147.32, *p* < 0.001, *F* = 69.86, *p* < 0.001). In addition, the interaction *E. bromicola* × salt was significant for proline, soluble sugar, and soluble protein contents (*F* = 93.48, *p* < 0.001, *F* = 7.35, *p* = 0.008, *F* = 18.05, *p* < 0.001, respectively, Table 1). These effects were related to *E. bromicola* inoculation at 300 mM. The proline content was 30% higher in E+ barley than in E– barley. Conversely, there was no significant difference between E+ and E– barley at a NaCl concentration of 100 mM (Figure 3A). *E. bromicola* inoculation had significant effects on the osmotic synthesis at different salt concentrations but significantly influenced the soluble sugar and soluble protein contents at NaCl concentrations of 100 and 300 mM (Figures 3B,C). Salt stress strongly increased the proline, soluble sugar, and soluble protein contents (Table 1).

### *Epichloë bromicola* changes metabolomes under salt stress

**Figure 4:**
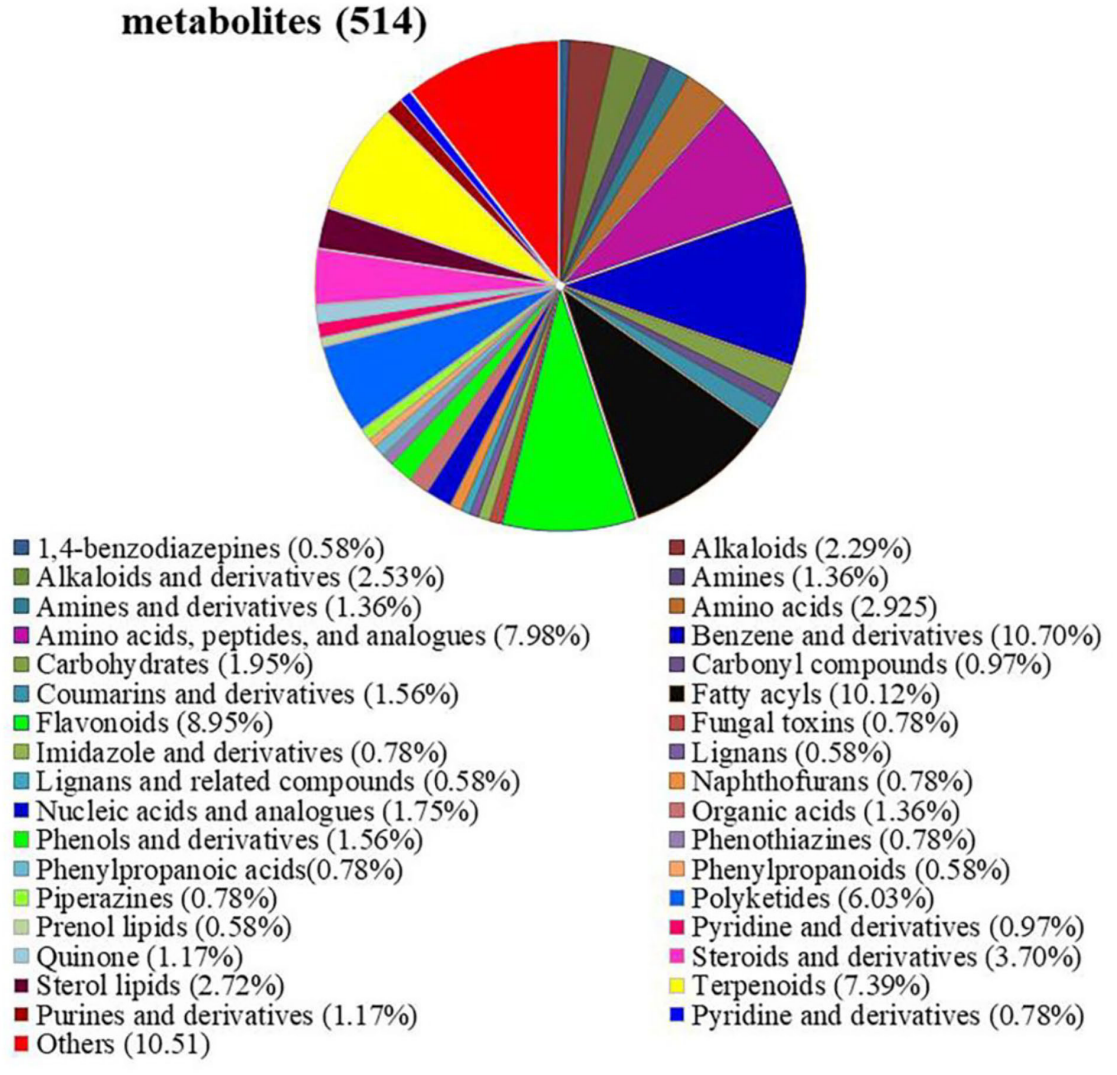
Detection of metabolites between E+ barley and E– barley under salt stress conditions. Pie chart showing the metabolite classification of barley inoculated with *E. bromicola* and subjected to NaCl concentrations of 0, 100, and 300 mM. The classification information in this figure is derived from HMDB and KEGG databases. Analysis and detection of metabolites according to LC-MS.

Untargeted metabolomic analysis was performed in E+ and E– barley plants subjected to different salt concentrations. In total, 514 metabolites were identified, including 34 different types of substances, such as amino acids, peptides and their analogs, carbohydrates, flavonoids, nucleic acids and their analogs, and imidazole and derivatives. Among them, the most abundant metabolites were benzene and derivatives (10.70%), fatty acyls (10.12%), flavonoids (8.95%), amino acids, peptides and their analogs (7.98%), terpenoids (7.39%), and polyketides (6.03%) (Figure 4, Supplementary Table S1).

**Figure 5:**
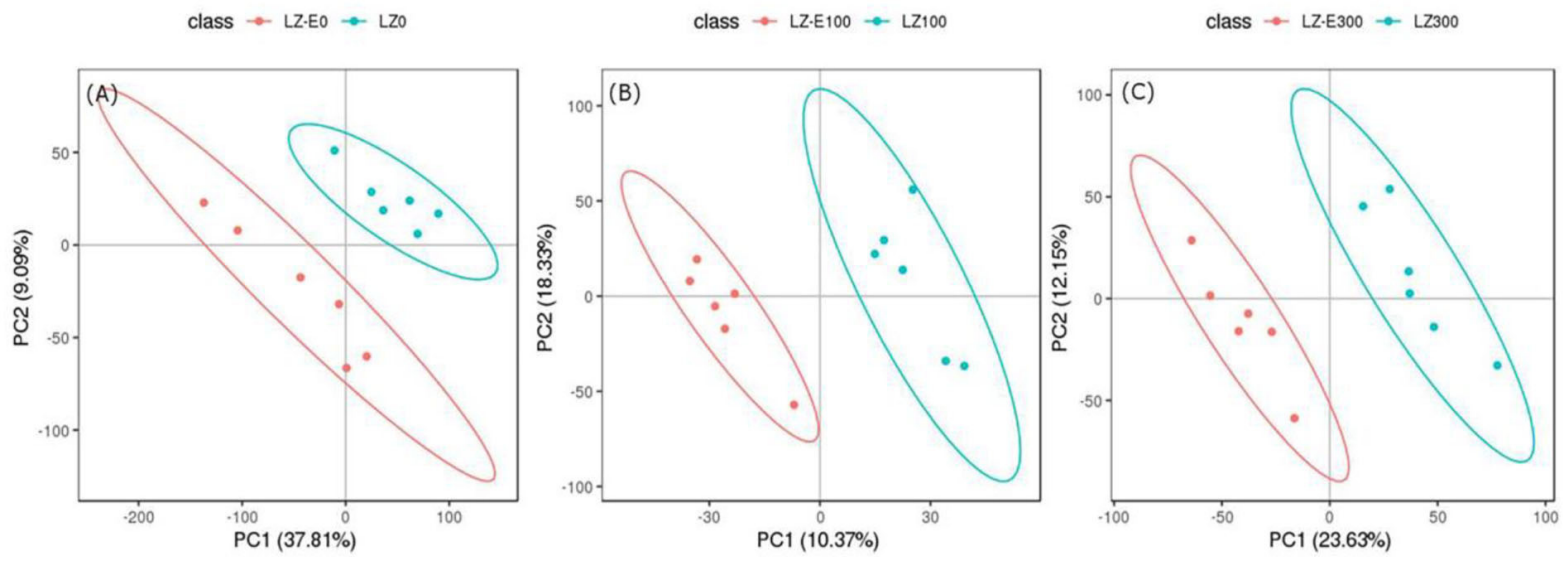
Analysis of metabolites between E+ barley and E– barley under salt stress conditions. PLS-DA analysis model score map. PCA score plot of metabolite analyses in barley samples for LC-MS data **(A–C)**. Red dots represent *E. bromicola*-inoculated barley at NaCl concentrations of 0, 100, and 300 mM (LZ-E0, LZ-E100, LZ-E300), and the blue dots represent *E. bromicola*-free barley at NaCl concentrations of 0, 100, and 300 mM (LZ0, LZ100, and LZ300). The horizontal axis is the first PC, and the vertical axis is the second PC. The number within parentheses is the score of the PC, indicating the ability of the PC to interpret the whole model.

**Figure 6:**
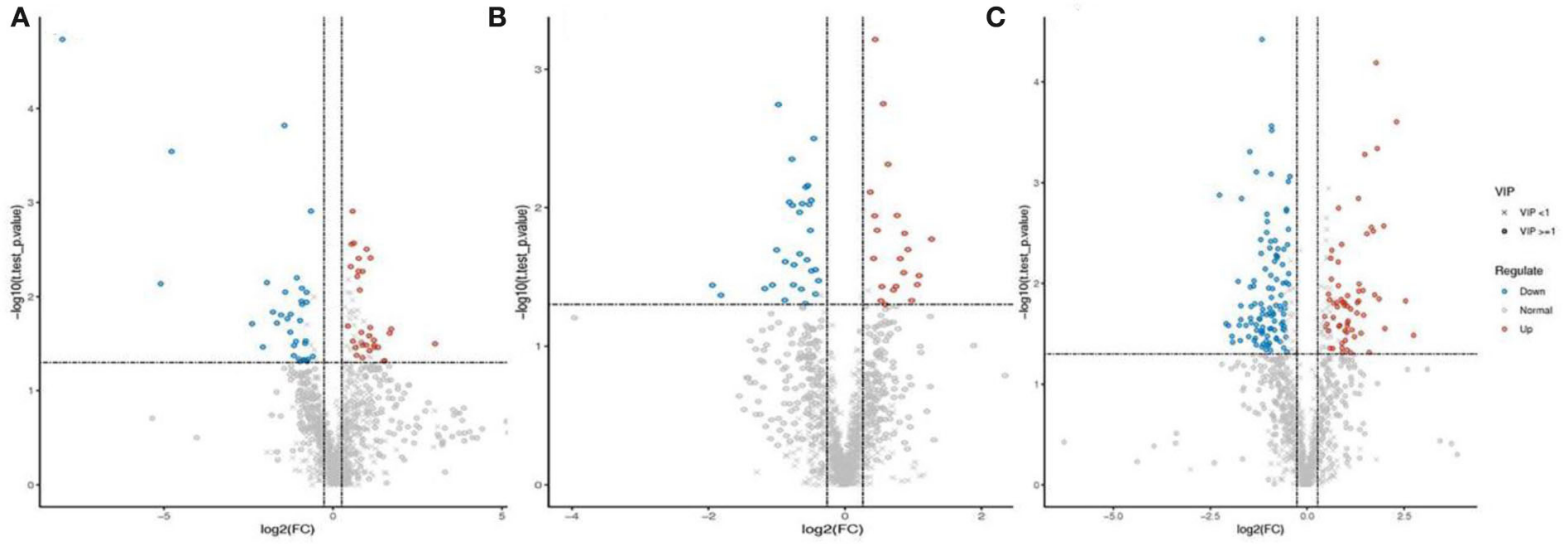
Differential metabolites between E+ barley and E– barley under salt stress conditions. Volcano plot of differential metabolites. Volcano plot of differential metabolites in barley samples for LC-MS data **(A–C)**. The volcano plot was used to visually display the selected differential ions. Blue dots are the downregulated significantly differential ions, the red dots are the upregulated significantly differential ions, the circles are the ions with VIP ≥1, the “ × ” represents ions with VIP <1, and the insignificant ions are marked gray. The measurement data are shown in Supplementary Tables S4–S6.

To identify differential metabolites between endophyte inoculation and salt stress, metabolites were selected using the variable importance in projection (VIP) value (VIP ≥ 1) from the PLS-DA model. The total number of differential metabolites was subjected to principal component analysis, with all metabolites showing a highly diverse metabolic profile among samples (Supplementary Figure S1). It was observed that *E. bromicola*-inoculated and salt samples were significantly different (Figures 5A–C). A total of 435 differential metabolites have been observed, among which 172 and 263 metabolites were upregulated and downregulated, respectively, by *E. bromicola* inoculation and salt stress (Table 2). Interestingly, the number of differential metabolites upregulated and downregulated by *E. bromicola* inoculation at a NaCl concentration of 300 mM (261) was higher than that at NaCl concentrations of 0 (100) and 100 mM (74). The number of differential metabolites upregulated and downregulated at a NaCl concentration of 100 mM was the lowest (Figures 6A–C; Table 2).

**Table 2:** Differentially expressed metabolites of E+ and E– barley under salt stress.

**Mode**	**Group**	**Total** **number of** **differential** **metabolites** **(level 1–3)**	**Number of** **upregulated** **metabolites**	**Number of** **downregulated** **metabolites**
Pos	LZ-E0:LZ0	67	32	35
Pos	LZ-E100:LZ100	50	21	29
Pos	LZ-E300:LZ300	173	65	108
Neg	LZ-E0:LZ0	33	17	16
Neg	LZ-E100:LZ100	24	12	12
Neg	LZ-E300:LZ300	88	25	63

The differential screening results of the comparative analysis group are shown in the table. Differential metabolite screening criteria: (1) the VIP values of the first two PCs of the PLS-DA model ≥1, (2) fold change ≥1.2 or ≤ 0.83, and (3) a p-value < 0.05.

**Figure 7:**
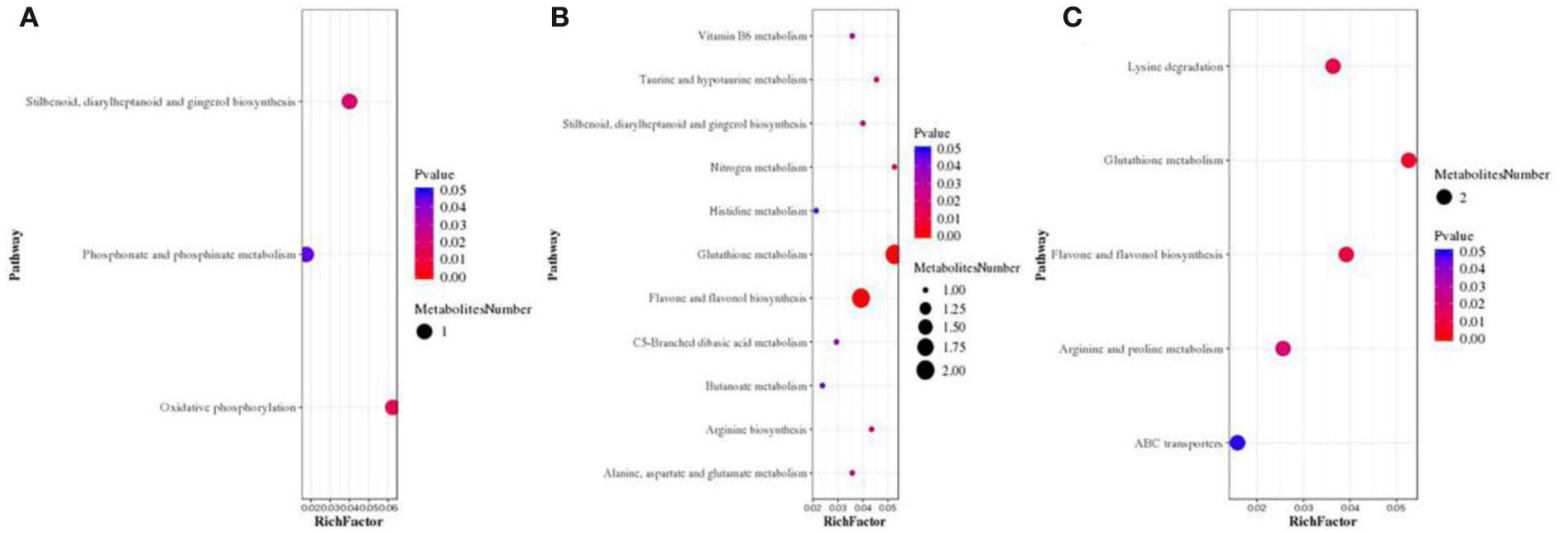
Screening for bubble charts of metabolic pathways involved in E+ barley and E– barley under salt stress conditions. Metabolic pathway enrichment analysis bubble chart. Metabolic pathway enrichment analysis of differential metabolites in barley samples for LC-MS data **(A–C)**. The *x*-axis Rich factor is the number of differential metabolites annotated in this pathway divided by all identified metabolites annotated in this pathway. The higher the value, the higher the ratio of differential metabolites annotated in this pathway. The dot size represents the number of differential metabolites annotated in this pathway. The measurement data are shown in Supplementary Table S7.

Subsequently, KEGG pathway enrichment analysis was examined to identify differences in metabolic pathways between E+ barley and E– barley plants under different salt stress conditions. The identified metabolites included phenylalanine, tyrosine, and tryptophan biosynthesis as well as oxidative phosphorylation at a NaCl concentration of 0 mM (Figure 7A; Supplementary Table S2). Glutathione metabolism, flavone and flavonol biosynthesis, lysine degradation, arginine and proline metabolism, ABC transporters, biosynthesis of amino acids, nitrogen metabolism, and 2-oxocarboxylic acid metabolism were significantly different (*p* < 0.05) between E+ barley and E– barley plants at NaCl concentrations of 100 mM (Figure 7B, Supplementary Table S3) and 300 mM (Figure 7C, Supplementary Table S4). L-glutamic acid is involved in numerous metabolic pathways, including glutathione metabolism, arginine and proline metabolism, ABC transporters, biosynthesis of amino acids, nitrogen metabolism, and carbon metabolism (Supplementary Table S5). These results indicate that the differences in metabolites between E+ and E– barley were strongly affected by salt stress.

## Discussion

### Effect of salt on endophyte–barley association

It is widely accepted that endophytic fungi play an important role in promoting host growth and alleviating both biotic and abiotic stresses of host plants. The results of this study support the hypothesis that *E. bromicola* increases the performance of barley under salt stress. Numerous studies have shown that endophytes promote host performance under salt stress (Redman et al., 2011; Card et al., 2015; Kumkum and Susan, 2015; Chen T. X. et al., 2021). This study found that inoculation with the endophyte *E. bromicola* promotes plant growth and physiological changes under several levels of salt stress. Salt stress induced substantial metabolomic changes in E+ and E– barley plants. Furthermore, previous work showed that wild barley infected with *E. bromicola* presented improved NaCl tolerance in pot experiments (Song et al., 2015; Wang et al., 2019; Chen T. X. et al., 2021). The results of the present study suggested that *E. bromicola* endophyte inoculation modified the salt-induced inhibition of barley growth.

### Effect of endophyte on barley

Extensive studies have been conducted to assess the potential of endophytes as inoculants for promoting plant tolerance under environment stresses and how they confer beneficial effects on plants (Oberhofer et al., 2014; Munns and Gilliham, 2015; Ali et al., 2018; Pereira et al., 2019). Possible mechanisms of growth promotion under salt stress include increasing the root biomass (Sabzalian and Mirlohi, 2010), producing phytohormones (Sekirov et al., 2010), improving photosynthesis (Leitão and Enguita, 2016), reducing the membrane injury rate (Chen X. L. et al., 2018), and producing various osmotic regulators (Gusain et al., 2015; Vurukonda et al., 2016). In this study, cultivated barley plants were inoculated with an asexual *E. bromicola* endophyte. The obtained results indicate that under salt stress, inoculation with this endophyte increased plant growth parameters, osmotic adjustment capacity, and leaf water content, as well as decreased the membrane injury rate. The accumulation of stress adaptation metabolites was higher in E+ barley plants than in E– barley plants. These experimental results showed that E+ barley has a higher salt resistance capability than E– barley in greenhouse conditions. Furthermore, *E. bromicola* infection in barley tissue showed no signs of pathogenesis and successfully transmitted within seeds. These results were consistent with findings of Llorens et al. (2019), who found that wheat plants inoculated with *Epichloë* endophytes showed improved performance under drought stress conditions. Thus, the results presented in this article provide evidence that cultivating barley that is inoculated with *E. bromicola* could be a suitable strategy to increase growth and improve the salt tolerance of barley.

### Potential of *E. bromicola* for improving cultivated barley growth

Breeding higher salt tolerance into cereals is one way to minimize the impact of saline soil and increase global food security. However, the salinity tolerance of crops remains poor. It has been confirmed that *E. bromicola* improves the growth of wild barley under salt stress conditions (Song et al., 2015; Wang et al., 2019). This study has experimentally demonstrated that *E. bromicola* can enhance the plant height, shoot biomass, and total biomass of cultivated barley, despite salt treatment. However, at a NaCl concentration of 300 mM, both E+ and E– plants exhibited a severe biomass reduction. Similar results were obtained by Rodriguez et al. (2008). Interestingly, at all salt treatment levels, the root biomass was higher in E+ barley plants than in E– barley plants. Similar positive effects were reported for wheat inoculated with the endophyte *S. implicatum* under drought stress (Llorens et al., 2019). The increased root biomass effect under salt stress is an important adaptive mechanism ensuring seedling establishment and allowing roots to increase their uptake of water and nutrients (Yamaguchi and Sharp, 2010; Shelden et al., 2013). This result demonstrates the potential of *E. bromicola* from wild barley plants for improving cultivated barley under salt stress conditions. This result suggests that *E. bromicola* can promote the growth of cultivated barley and can help to cultivate barley under salt stress. The observed growth enhancement of wheat plants has been attributed to the inoculation with endophytes that were isolated from wild cereals under water-limited conditions (Llorens et al., 2019).

### Increased leaf chlorophyll content and RWC, and decreased membrane injury

The chlorophyll content, RWC, and membrane injury rate of plants are commonly used stress indicators (Ueda et al., 2003; Chaves et al., 2009; Mehta et al., 2010; Füzy et al., 2019). Recent studies proposed that changes in the chlorophyll content, RWC, and REC may be affected by endophytes in plants growing under stress (Zhang et al., 2019). The chlorophyll content of E+ and E– barley plants were not significantly different under the same salt treatments. This result was not consistent with studies by (Chen T. X. et al., 2018), who found that *E. bromicola* infection of wild barley plants resulted in an improved chlorophyll content under salt stress. The results of the present study provided evidence that infection by an *E. bromicola* endophyte may elevate the leaf RWC and decrease the REC in barley, notably at a NaCl concentration of 100 mM. *E. bromicola* can decrease the membrane injury rate in E+ plants, which increases salt tolerance. A previous study found that inoculation with *Pseudomonas* sp. significantly decreased the REC of ryegrass (*Lolium perenne* L. cv. Esquire) under high salt stress (He et al., 2018). The increased chlorophyll content and RWC in leaves of E+ plants may be related to the ability of plants to improve their salt tolerance. These results suggested that the endophyte has potential for enhancing salt tolerance in barley.

### Effects of *E. bromicola* on the osmotic adjustment capacity of cultivated barley

With constant salt stress at a NaCl concentration of 300 mM, E– barley wilted severely within 21 days. By contrast, E+ barley also wilted slightly within the same time. Significant differences in the soluble sugar, soluble protein, and proline contents were observed between E+ barley and E– barley at a NaCl concentration of 300 mM. These are important osmotic adjustment compounds and may reflect differences between E+ and E– barley in their response and adaptation to environmental stresses. This study showed that E+ barley is more resistant to salt stress, which confers an enhanced osmotic synthesis capacity. It has been previously demonstrated that *E. bromicola* infection plays a role in increasing the proline content in wild barley under salt stress (Chen et al., 2019a). Previous studies have found that the presence of endophytes can produce osmolytes in response to salt stress, many of which may protect apical meristems and can maintain the host cell turgor (Hamilton and Bauerle, 2012; Giauque et al., 2018). Furthermore, these osmotic adjustment compounds have been shown to be important for cell turgor and allow maintenance of water uptake under stress conditions (Chen and Jiang, 2010). *E. bromicola* enhanced the osmotic adjustment capacity of E+ plants compared with E– plants, and E+ plants remained in a better physiological state under salt stress conditions.

### Impact of endophyte symbiosis on metabolomes

In the present study, growth and physiological parameter changes of E+ and E– barley plants were compared under NaCl salinity. Furthermore, the potential mechanisms underlying the enhanced salt stress tolerance were explored. The metabolic profiles of E+ barley and E– barley plants subjected to salinity stress were compared under different salt conditions. Different metabolites of E+ and E– barley plants were observed under different salt stress conditions. For both E+ and E– barley plants, more metabolites were downregulated under 300 mM salt conditions than under 0 and 100 mM salt conditions. This indicates that 300 mM salt stress stimulated the physiological metabolism of barley plants. Under the 100 mM salt condition, the number of upregulated metabolites was lowest in E+ barley and E– barley, while under the 300 mM salt condition, the number of upregulated metabolites was highest, reflecting the adaptability of barley plants to high salt stress. Previous studies on maize showed that the abundance of metabolites differed under salt stress, and, while 30% were downregulated, 70% were upregulated (Liang et al., 2021). The results of the present study suggested that metabolites are upregulated or downregulated in response to salt stress.

It was also observed that the metabolites covered a wide range of metabolite classes, including fatty acyls, flavonoids, and carbohydrates. Fatty acids play an important role in signal transduction pathways, as cellular fuel sources, as part of the composition of hormones and lipids, and in the modification of proteins. Previous studies have shown that fatty acids play important roles in plant tolerance under salt stress (Rodriguez et al., 2008). In this study, 52 fatty acid metabolites have been identified, which accumulated differently between E+ barley and E– barley plants, indicating that fatty acids may play important roles in barley salt tolerance. Flavonoids are important secondary metabolites in plants, which play an important role in plant growth, have many biological functions, and respond to various environmental stresses, for example, salt stress and drought stress (Bian et al., 2018; Yang et al., 2019). The rate of flavonoid accumulation was higher in leaves of barley plants under salt treatment. Therefore, *E. bromicola* seemed to overcompensate the salt-induced inhibition of plant metabolic activity. It has been shown that flavonoids convey protective properties through strong antioxidant activity (Agati et al., 2012), and flavonoid concentrations increased significantly in *Ginkgo biloba* seedlings at a NaCl concentration of 100 mM (Xu et al., 2020).

In addition to the observed osmotic adjustment compounds (soluble sugar, soluble protein, and proline contents), KEGG enrichment analysis identified metabolic pathways between E+ and E– barley plants under different salt stress conditions. Importantly, the accumulation of metabolites with osmotic protectant ability (such as arginine and proline) was observed, and the concentrations of these compounds remained significantly affected at a high salt concentration. Among the compounds enriched in E+ and E– barley plants, 6, 33, and 13 specialized plant pathways were found to besignificantly different (*p* < 0.05) under different NaCl stresses (0, 100, and 300 mM, respectively). Fardus et al. (2021) reported that L-glutamic acid application enhanced the survival of lentil seedlings and the contents of chlorophyll, and seedlings accumulated more proline under salt stress. Glutamic acid, as an amino acid and signaling molecule, plays an important part in the adaptation to a stressful environment (Qiu et al., 2020). L-glutamic acid was strongly accumulated in arginine and proline metabolism, nitrogen metabolism, and carbon metabolism. Recently, Sadak et al. (2015) also found that L-glutamic acid application enhanced the biomass of faba bean plants under salt stress conditions. In addition, the results of this study provide further evidence that the increasing physiology metabolism of barley plays an important role in their ability to survive saline conditions. These results partially explain why *E. bromicola* inoculation renders barley with better salt tolerance.

## Conclusion

In summary, barley plants inoculated with *E. bromicola* showed increased levels of salt stress response compared with uninoculated barley plants. *E. bromicola* had positive effects on E+ plant growth and improved their physiology status under salt stress. This study showed that *E. bromicola*, isolated from congener wild relatives of barley, had potential for alleviating salt stress in cultivated barley. Through inoculation, endophytic fungi can be exploited to produce hardier germplasm, which is a promising emerging technology to alleviate problems caused by salt stress.

## Data availability statement

The original contributions presented in the study are included in the article/Supplementary material, further inquiries can be directed to the corresponding author.

## Author contributions

ZW and JL planned and designed the experiment. ZW analyzed the data and wrote the manuscript. CL and JW reviewed and edited the manuscript. All authors contributed to the editing of the manuscript and approved the final version.

## Funding

This research was financially supported by the Doctor Foundation of Gansu Academy of Agricultural Sciences (2022GAAS62), the National Basic Research Program of China (2014CB138702), the Natural Science Foundation of China (31971756, 31372366), and the Program for Changjiang Scholars and Innovative Research Team in the University of China (IRT17R50). The authors are also grateful for support from the USDA-NIFA Multistate Project W4147 and the New Jersey Agricultural Experiment Station.

## Conflict of interest

The authors declare that the research was conducted in the absence of any commercial or financial relationships that could be construed as a potential conflict of interest.

## Publisher's note

All claims expressed in this article are solely those of the authors and do not necessarily represent those of their affiliated organizations, or those of the publisher, the editors and the reviewers. Any product that may be evaluated in this article, or claim that may be made by its manufacturer, is not guaranteed or endorsed by the publisher.
