# Supplementary material for: Epichloë bromicola from wild barley improves salt-tolerance of cultivated barley by altering physiological responses to salt stress
Source: Front Microbiol. 2022 Nov 24;13:1044735. doi: 10.3389/fmicb.2022.1044735 (PMC9730248; doi:10.3389/fmicb.2022.1044735)
Supplement: Supplementary file 1 [file Data_Sheet_1.docx]

Supporting Information

Article title: **Improved salt-tolerance from a novel barley- fungal endophyte association**

Authors: Zhengfeng Wang ^1^, Jing Liu^2^, James F. White^3^, Chunjie Li ^2,4*^

The following Supporting Information is available for this article:

**Fig. S1** PCA model score map.

**Table S1** Classification of 514 metabolites.

**Table S2** Statistics of Differential Metabolites (The numbers of differential metabolites were upregulated and downregulated at NaCl concentrations 0 mM under LC-MS.

**Table S3** Statistics of Differential Metabolites (The numbers of differential metabolites were upregulated and downregulated at NaCl concentrations 100 mM under LC-MS.

**Table S4** Statistics of Differential Metabolites (The numbers of differential metabolites were upregulated and downregulated at NaCl concentrations 300 mM under LC-MS.

**Table S5** The metabolic pathway enrichment analysis of differential metabolites.


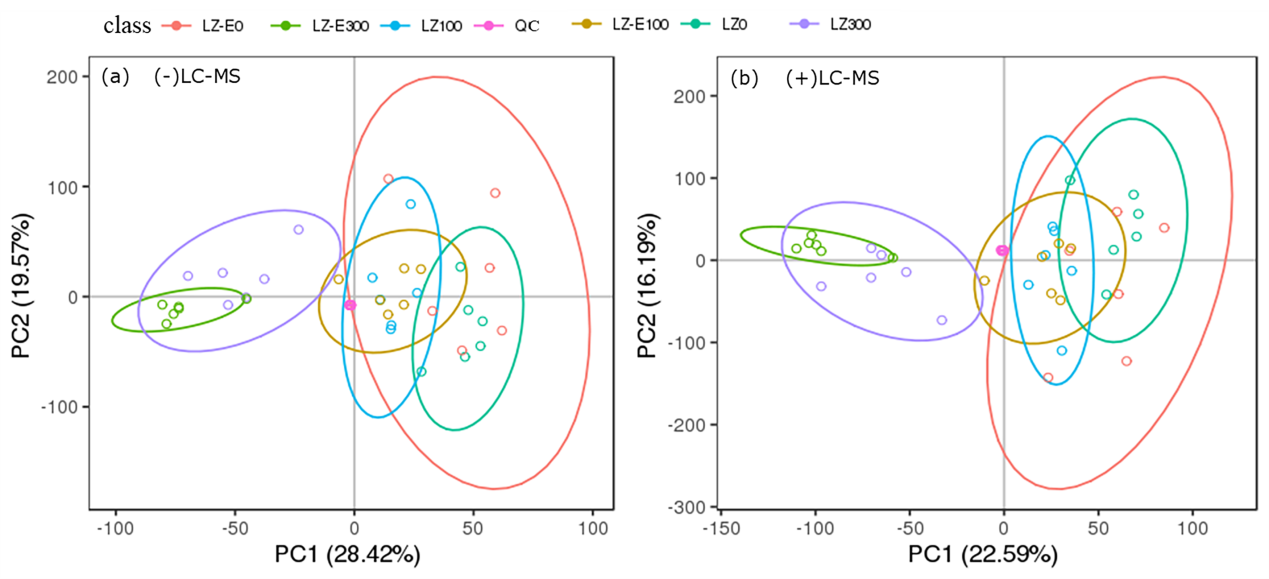


**Fig S1** PCA score plot of metabolite analyses in barley samples for LC-MS data . The abscissa represents the first principal component PC1, the ordinate represents the second principal component PC2, and the ellipse in the PCA score graph is 95% confidence interval. Each dot represents a sample, with different colors for different groups, the red dots represent *E. bromicola* - inoculated barley under 0 mM NaCl (LZ-E0), the blue dots represent *E. bromicola* - free barley under 0 mM NaCl (LZ0), the brown dots represent *E. bromicola* - inoculated barley under 100 mM NaCl (LZ-E100), the dark bule dots represent *E. bromicola* - free d barley under 100 mM NaCl (LZ100), the green dots represent *E. bromicola* - inoculated barley under 300 mM NaCl (LZ-E300), the orchid dots represent *E. bromicola* - free barley under 300 mM NaCl (LZ300), the purple dots represent QC. The number in parentheses is the score of the PC, indicating the proportion that can synthesize the original information
